# Association between carnitine deficiency and the erythropoietin resistance index in patients undergoing peritoneal dialysis: a cross-sectional observational study

**DOI:** 10.1080/0886022X.2020.1719847

**Published:** 2020-01-31

**Authors:** Shohei Kaneko, Keiji Hirai, Junki Morino, Saori Minato, Katsunori Yanai, Yuko Mutsuyoshi, Hiroki Ishii, Momoko Matsuyama, Taisuke Kitano, Mitsutoshi Shindo, Akinori Aomatsu, Haruhisa Miyazawa, Yuichiro Ueda, Kiyonori Ito, Susumu Ookawara, Yoshiyuki Morishita

**Affiliations:** Division of Nephrology, First Department of Integrated Medicine, Saitama Medical Center, Jichi Medical University, Saitama, Japan

**Keywords:** Anemia, carnitine deficiency, erythropoietin resistance index, peritoneal dialysis

## Abstract

Carnitine deficiency contributes to developing various pathological conditions, such as cardiac dysfunction, muscle weakness, and erythropoietin-resistant anemia in patients undergoing hemodialysis. However, a conclusion has not been reached concerning the prevalence and the effect of carnitine deficiency in patients undergoing peritoneal dialysis (PD). In this study, the prevalence of carnitine deficiency and the clinical factors associated with carnitine deficiency were investigated in 60 patients undergoing PD. The median age of the patients was 62.5 years (52.5–72.5 years), the proportion of male sex was 44/60 (73.3%), and the median PD period was 24 months (12–45 months). Carnitine deficiency (acyl carnitine/free carnitine ratio >0.4) was detected in 56/60 (93%) patients. Multiple regression analysis showed that the erythropoietin resistance index was independently associated with carnitine deficiency (β = 0.283, *p* = 0.04). These results suggest that carnitine plays pivotal roles in hematogenesis in patients undergoing PD.

## Introduction

Carnitine is an essential amino-acid derivative and plays pivotal roles in fatty-acid metabolism in skeletal muscle and cardiac muscle [1–3]. Carnitine is present in two forms in the body, acyl carnitine and free carnitine, and the sum of them is defined as total carnitine [4]. Free carnitine is converted to acylcarnitine by binding to an acyl residue. Additionally, acyl carnitine functions as a transporter of fatty acids to mitochondria and as a scavenger of excess and harmful acyl residues in cells [4]. A total of 75% of carnitine in the body is obtained by dietary intake, such as red meats, and the remaining 25% is biosynthesized by the kidney and liver [5,6]. In healthy individuals, most of the free carnitine is re-reabsorbed in the kidney, and acyl carnitine is preferentially excreted into urine. Carnitine homeostasis in the body is maintained by this mechanism.

Previous studies have been reported a high prevalence of carnitine deficiency in patients undergoing hemodialysis [1,5,7]. Carnitine deficiency in patients undergoing hemodialysis is defined as a low free carnitine level (<20 μmol/L) or imbalance between acyl carnitine and free carnitine (acyl carnitine/free carnitine >0.4) [7,8]. The causes of carnitine deficiency in patients undergoing hemodialysis are considered to be insufficient carnitine intake, decreased biosynthesis, removal by hemodialysis, and loss of preferential renal excretion of acyl carnitine [9,10].

Carnitine deficiency is associated with various pathological conditions, including anemia, cardiac dysfunction, and muscle weakness in patients undergoing hemodialysis [2,11–16]. The National Kidney Foundation has proposed that carnitine deficiency in patients undergoing hemodialysis is a dialysis-related carnitine disorder in a conference report [17]. They recommend carnitine supplementation for patients undergoing hemodialysis who have erythropoietin-resistant anemia, hypotension during hemodialysis sessions, cardiac dysfunction, and muscle weakness [17]. Therefore, detection of carnitine deficiency and appropriate carnitine supplementation are important for treating patients undergoing hemodialysis. There have also been several reports on carnitine deficiency and carnitine supplementation in patients undergoing peritoneal dialysis (PD) [18–22]. However, these studies had small sample sizes, leading to contradictory conclusions [18–22]. Although there appear to be similar risks for carnitine deficiency in patients undergoing PD as those undergoing hemodialysis, there are no established diagnostic criteria for carnitine deficiency and no guideline for carnitine supplementation in patients undergoing PD at this time. Therefore, studies on the prevalence and clinical effects of carnitine deficiency in patients undergoing PD are still required. In this study, we aimed to clarify the prevalence and clinical effect of carnitine deficiency in patients undergoing PD.

## Materials and methods

### Study design

We performed an observational, retrospective study. We examined patients’ characteristics and conducted a cross-sectional analysis of the prevalence of carnitine deficiency and factors associated with carnitine deficiency in patients undergoing PD.

### Patients

This study was retrospectively conducted at Saitama Medical Center, Jichi Medical University. We investigated outpatients who underwent PD between January 2018 and December 2018. Inclusion criteria were as follows: (i) patients with end-stage renal failure undergoing PD; (ii) patients who had carnitine levels measured; and (iii) age > 18 years. Exclusion criteria were as follows: (i) patients who were receiving carnitine supplementation; (ii) acute kidney injury; (iii) patients who had undergone renal transplantation; and (iv) no consent to participate in this study. All of the patients were treated in accordance with the 2009 Japanese Society for Dialysis Therapy Guideline for PD and the 2015 Japanese Society for Dialysis Therapy Guideline for Renal Anemia in Chronic Kidney Disease [23,24]. This study was approved by the Saitama Medical Center, Jichi Medical University Ethics committee (DAI-RIN 15-34) and was conducted in accordance with the Declaration of Helsinki. Because this study was observational and retrospective, it was exempt from obtaining a consent form from individual patients and it was replaced with opt-out.

### Data collection

All of the data were collected retrospectively from medical records: information of age, sex, body mass index, underlying diseases (diabetes mellitus, hepatic disease, cardiac diseases), PD period, hemodialysis combination, and PD modality. In this study, the modality of PD was initially classified as two types as follows. Continuous ambulatory PD (CAPD) is a method of changing PD fluids manually during the daytime with or without maintaining PD fluid retention during the nighttime. Automated PD (APD) is a method of changing PD fluids with automated systems [25–28]. APD was then further classified as nocturnal intermittent PD (NIPD) and continuous cyclic PD (CCPD). NIPD is APD during the nighttime without maintaining PD fluid retention during the daytime. CCPD is APD during the nighttime with maintenance of PD fluid retention during the daytime [25–28]. We also collected data on total weekly Kt/V urea, peritoneal weekly Kt/V urea, renal weekly Kt/V urea, 4-h dialysate/plasma creatinine, erythropoietin resistance index, ejection fraction, and laboratory markers, including carnitine levels. Diabetes mellitus was defined as hemoglobin A1c ≥ 6.5% or use of oral hypoglycemic agents and/or insulin therapy. Hepatic disease was defined as chronic liver injury, such as hepatitis and cirrhosis. Cardiac disease was defined as coronary artery disease or arrhythmia. Hemodialysis combination was defined as the combined treatment of hemodialysis once a week in addition to PD. Peritoneal weekly Kt/V urea was an indicator of efficacy of PD and renal weekly Kt/V was an indicator of residual renal function. Their summation was defined as total weekly Kt/V urea [29]. Four-hour dialysate/plasma creatinine is known as an indicator of peritoneal permeability [30].

### Laboratory markers

Total carnitine and free carnitine levels were measured by a clinical chemistry laboratory (SRL, Tokyo, Japan) using the enzyme cycling method. This is an established method for accurately measuring carnitine levels [31]. Acyl carnitine levels were calculated by total carnitine minus free carnitine levels [4]. Other blood and PD dialysate fluid parameters were determined by the Department of Clinical Laboratory, Saitama Medical Center.

### Definition of carnitine deficiency

In accordance with previous studies, the diagnostic criterion for carnitine deficiency in this study was a low free carnitine level (<20 μmol/L) or a high acyl carnitine/free carnitine ratio (>0.4) [7,8].

### Definition of the erythropoietin resistance index

The resistance to the erythropoiesis-stimulating agent of renal anemia was evaluated by the erythropoietin resistance index [2,12]. The erythropoietin resistance index was defined as the average weekly dose of recombinant human erythropoietin (IU)/body weight (kg)/hemoglobin (g/dL) [2,12]. For dose conversion of recombinant human erythropoietin, the ratio of recombinant human erythropoietin darbepoetin alfa or epoetin beta pegol was converted to 200:1 [32].

### Statistical analysis

The normality of distribution of the sample was determined by the Shapiro–Wilk test. For measurement data, quantitative variables are expressed as mean ± standard deviation for a normal distribution and as median and interquartile range for a non-normal distribution. Categorical variables are described as frequency and percentage. Fisher’s exact test was used for comparison of frequency. Continuous variables were compared using the Wilcoxon rank-sum test and one-way analysis of variance. Tukey’s multiple comparison test was used for multiple comparisons. Univariate and multiple regression analyses were performed to investigate the factors associated with the acyl carnitine/free carnitine ratio and erythropoietin resistance index. Multiple regression analysis was performed using the parameters that showed a significant correlation in univariate analysis. Patients were divided into two groups, with the median erythropoietin resistance index, and related parameters were compared. Statistical analysis was performed using JMP (SAS Institute Inc., Cary, NC, USA). Values of *p* < 0.05 were considered statistically significant.

## Results

### Clinical characteristics of the patients

Sixty patients met the criteria and were enrolled in this study. Table 1 shows the clinical characteristics of the study population (*n* = 60). The median age of the patients was 62.5 years (interquartile range: 52.5–72.5). Forty-four (73.3%) men were enrolled in the study. The median PD period was 24 months (12–45 months). Diabetes mellitus, hepatic disease, and cardiac disease were found in 23 (38.3%), two (3.3%) and 24 (40.0%) patients, respectively. Continuous ambulatory PD, nocturnal intermittent PD and continuous cyclic PD were selected as PD modalities for eight (13.3%), 21 (35.0%), and 31 (51.7) patients, respectively. The median total carnitine, free carnitine, and acyl carnitine levels were 45.2 µmol/L (36.6–52.7 µmol/L), 27.5 µmol/L (22.5–34.0 µmol/L), and 16.6 µmol/L (13.3–19.3 µmol/L), respectively. The median acyl carnitine/free carnitine ratio was 0.5 (0.5–0.7). The mean erythropoietin resistance index was 7.0 ± 4.4. Table 2 shows the prevalence of carnitine deficiency in patients undergoing PD. A total of 8% (5/60) of patients undergoing PD had low free carnitine level and 93% (56/60) of patients undergoing PD had a high acyl carnitine/free carnitine ratio. All patients with low free carnitine level had a high acyl carnitine/free carnitine ratio. The prevalence of carnitine deficiency was not different between the PD group and PD + HD combination group (Table 3). The prevalence of carnitine deficiency was also not different among different PD modalities (CAPD, NIPD, and CCPD) (Table 4).

**Table 1.** Characteristics of the study population (*n* = 60).

Variable	Mean ± SD or median [IQR]	Number (%)
Age (years)	62.5 [52.5–72.5]	
Male sex		44 (73.3)
Body mass index (kg/m^2^)	23.2 [20.8–25.3]	
Diabetes mellitus		23 (38.3)
Hepatic disease		2 (3.3)
Cardiac disease		24 (40.0)
PD period (months)	24 [12–45]	
Hemodialysis combination		13 (21.7)
PD modality: CAPD		8 (13.3)
PD modality: APD (NIPD)		21 (35.0)
PD modality: APD (CCPD)		31 (51.7)
White blood cells (10^3^/µL)	6.24 [5.13–7.47]	
Red blood cells (10^4^/µL)	350.1 ± 46.2	
Hemoglobin (g/dL)	10.8 ± 1.3	
Platelets (×10^4^/µL)	22.9 ± 7.5	
Total protein (g/dL)	6.3 ± 0.5	
Albumin (g/dL)	3.3 ± 0.5	
Blood urea nitrogen (mg/dL)	52.0 [48.0–62.8]	
Creatinine (mg/dL)	10.9 ± 3.2	
C-reactive protein (mg/dL)	0.15 [0.04–0.48]	
Uric acid (mg/dL)	5.9 [5.0–7.2]	
Sodium (mEq/L)	138.2 ± 4.0	
Potassium (mEq/L)	4.4 [3.8–5.1]	
Chloride (mEq/L)	99.8 ± 5.0	
Corrected calcium (mg/dL)	9.1 ± 0.6	
Phosphorus (mg/dL)	5.6 [4.6–6.0]	
Magnesium (mg/dL)	2.1 [1.8–2.5]	
Zinc (µg/dL)	60.5 [52.0–68.0]	
Copper (µg/dL)	95.5 ± 20.8	
Iron (µg/dL)	74.5 [52.0–95.8]	
Ferritin (mg/mL)	134 [85–254]	
Transferrin saturation (%)	30.9 [21.2–39.2]	
Vitamin B12 (pg/mL)	384 [275–734]	
Folate (ng/mL)	4.5 [3.5–6.3]	
HbA1c (%)	5.6 [5.0–6.1]	
Total carnitine (µmol/L)	45.2 [36.6–52.7]	
Acyl carnitine (µmol/L)	16.6 [13.3–19.3]	
Free carnitine (µmol/L)	27.5 [22.5–34.0]	
Acyl carnitine/free carnitine ratio	0.5 [0.5–0.7]	
Total cholesterol (mg/dL)	178 ± 47	
HDL cholesterol (mg/dL)	46 [35–54]	
LDL cholesterol (mg/dL)	97 [69–122]	
Triglycerides (mg/dL)	124 [87–181]	
Intact PTH (pg/dL)	231 [144–336]	
ERI (IU/week/kg/(g/dL))	7.0 ± 4.4	
Total Kt/V urea	1.61 [1.40–1.83]	
Peritoneal Kt/V urea	1.07 [0.83–1.36]	
Renal Kt/V urea	0.44 [0.25–0.82]	
D/P Cre	0.66 ± 0.14	
Ejection fraction (%)	64 [59–68]	

SD: standard deviation; IQR: interquartile range; PD: peritoneal dialysis; CAPD: continuous ambulatory peritoneal dialysis; APD: automated peritoneal dialysis; NIPD: nocturnal intermittent peritoneal dialysis; CCPD: continuous cyclic peritoneal dialysis; HbA1c: hemoglobin A1c; HDL: high-density lipoprotein; LDL: low-density lipoprotein; PTH: parathormone; ERI: erythropoietin resistance index; D/P Cre: 4-h dialysate/plasma creatinine.

**Table 2.** Prevalence of carnitine deficiency in patients undergoing PD (*n* = 60).

Carnitine levels	Number (%)
Free carnitine <20 μmol/L	5 (8)
Acyl carnitine/free carnitine ratio >0.4	56 (93)

PD: peritoneal dialysis.

**Table 3.** Comparison of the prevalence of carnitine deficiency (HD combination) (*n* = 60).

	PD alone group (*n* = 47)	PD + HD combination group (*n* = 13)	
Carnitine levels	Number (%)		*p* Value
Free carnitine <20 μmol/L	5 (11)	0 (0)	0.58
Acyl carnitine/free carnitine ratio >0.4	43 (91)	13 (100)	0.57

HD: hemodialysis; PD: peritoneal dialysis.

**Table 4.** Comparison of the prevalence of carnitine deficiency (PD modality) (*n* = 60).

	CAPD (*n* = 21)	APD (NIPD) (*n* = 8)	APD (CCPD) (*n* = 31)	
Carnitine levels	Number (%)			*p* Value
Free carnitine <20 μmol/L	3 (14)	0 (0)	2 (6)	0.41
Acyl carnitine/free carnitine ratio >0.4	20 (95)	7 (88)	29 (94)	0.76

PD: peritoneal dialysis; CAPD: continuous ambulatory peritoneal dialysis; APD: automated peritoneal dialysis; NIPD: nocturnal intermittent peritoneal dialysis; CCPD: continuous cyclic peritoneal dialysis.

### Factors associated with carnitine deficiency in patients who underwent PD

**Figure 1.**
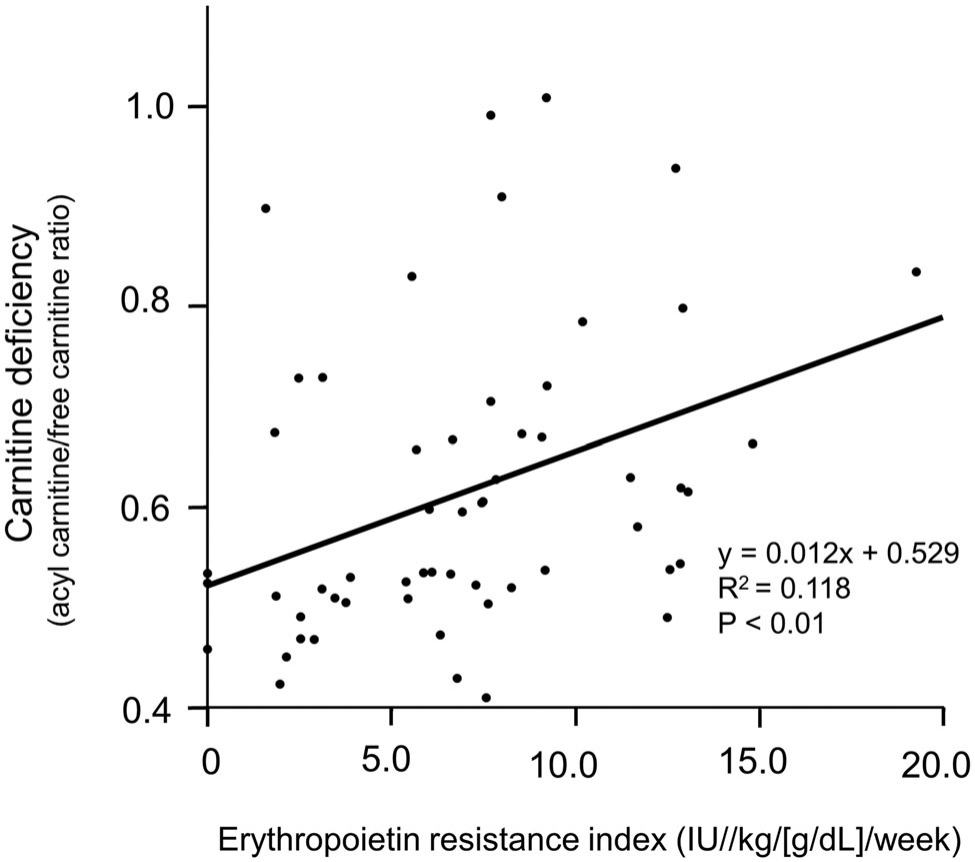
Significant correlation between carnitine deficiency (acyl carnitine/free carnitine ratio) and the erythropoietin resistance index in patients who underwent peritoneal dialysis.

The correlations between the acyl carnitine/free carnitine ratio and the clinical factors of patients undergoing PD with carnitine deficiency (*n* = 56) are shown in Table 5. In univariate analysis, the PD period (*p* = 0.03, β = 0.294) and erythropoietin resistance index (*p* < 0.01, β = 0.343) were significantly correlated with carnitine deficiency. In multiple regression analysis, the erythropoietin resistance index (*p* = 0.04, β = 0.293) was significantly correlated with carnitine deficiency (Table 5 and Figure 1). The correlations between the erythropoietin resistance index and clinical factors, including free carnitine level and the acyl carnitine/free carnitine ratio, in patients who underwent PD with carnitine deficiency (*n* = 56) are shown in Table 6. Univariate and multivariate analyses showed that free carnitine level and the acyl carnitine/free carnitine ratio were significantly correlated with the erythropoietin resistance index in patients undergoing PD with carnitine deficiency. Additionally, we compared these factors between a group of patients who had a low erythropoietin resistance index and a group who had a high erythropoietin resistance index. No factors, except for free carnitine levels and the acyl carnitine/free carnitine ratio, were significantly different between these two groups (Table 7).

**Table 5.** Factors associated with the acyl carnitine/free carnitine ratio in patients undergoing PD with carnitine deficiency (*n* = 56).

Variables	Univariate analysis		Multivariate analysis	
	β	*p* Value	β	*p* Value
Age	−0.143	0.29		
Sex male	−0.095	0.49		
Body mass index	0.045	0.74		
Diabetes mellitus	0.019	0.89		
Liver disease	0.100	0.47		
Heart disease	0.151	0.26		
PD period	0.294	0.03	0.213	0.11
Hemodialysis combination	0.102	0.46		
PD modality: CAPD	0.143	0.28		
PD modality: APD (NIPD)	0.179	0.17		
PD modality: APD (CCPD)	−0.014	0.91		
White blood cells	0.182	0.18		
Red blood cells	−0.101	0.46		
Hemoglobin	−0.187	0.17		
Platelets	0.192	0.16		
Total protein	0.080	0.56		
Albumin	−0.006	0.96		
Blood urea nitrogen	0.101	0.46		
Creatinine	0.254	0.06		
C-reactive protein	0.130	0.34		
Uric acid	0.234	0.08		
Sodium	−0.125	0.36		
Potassium	0.144	0.29		
Chloride	−0.151	0.27		
Corrected calcium	0.136	0.32		
Phosphorus	−0.013	0.92		
Magnesium	0.072	0.60		
Zinc	0.164	0.23		
Copper	0.091	0.50		
Iron	−0.164	0.23		
Ferritin	0.236	0.08		
Transferrin saturation	−0.185	0.17		
Vitamin B12	−0.352	0.13		
Folate	0.111	0.65		
Blood glucose	−0.085	0.53		
HbA1c	−0.143	0.29		
Total cholesterol	0.071	0.60		
HDL cholesterol	0.051	0.71		
LDL cholesterol	0.125	0.36		
Triglycerides	−0.133	0.33		
Intact PTH	−0.061	0.66		
ERI	0.343	<0.01	0.283	0.04
Total Kt/V urea	−0.084	0.54		
Peritoneal Kt/V urea	0.153	0.26		
Renal Kt/V urea	−0.106	0.43		
D/P urea	0.111	0.42		
Ejection fraction	−0.062	0.65		

PD: peritoneal dialysis; CAPD: continuous ambulatory peritoneal dialysis; APD: automated peritoneal dialysis; NIPD: nocturnal intermittent peritoneal dialysis; CCPD: continuous cyclic peritoneal dialysis; HbA1c: hemoglobin A1c; HDL: high-density lipoprotein; LDL: low-density lipoprotein; PTH: parathormone; ERI: erythropoietin resistance index; D/P Cre: 4-h dialysate/plasma creatinine.

**Table 6.** Factors associated with the ERI in patients undergoing PD with carnitine deficiency (*n* = 56).

Variables	Univariate analysis		Multivariate analysis	
	β	*p* Value	β	*p* Value
Age	−0.235	0.08		
Sex male	0.040	0.78		
Body mass index	−0.119	0.38		
Iron	−0.164	0.23		
Transferrin saturation	−0.196	0.15		
Ferritin	0.251	0.06		
C-reactive protein	0.171	0.21		
Corrected calcium	0.238	0.08		
Phosphorus	−0.116	0.40		
Intact PTH	−0.089	0.52		
Vitamin B12	−0.21	0.41		
Folate	0.153	0.56		
Albumin	−0.245	0.07		
Zinc	0.123	0.36		
Free carnitine	−0.341	0.01	−0.268	0.04
Acyl carnitine/free carnitine ratio	0.343	<0.01	0.271	0.04

ERI: erythropoietin resistance index; PD: peritoneal dialysis; Intact PTH: intact parathormone.

**Table 7.** Comparison of clinical parameters between the low ERI group and the high ERI group in patients undergoing PD with carnitine deficiency (*n* = 56).

	Low ERI group (*n* = 28)	High ERI group (*n* = 28)	
Carnitine status	Mean ± SD or median [IQR] or number (%)		*p* Value
Age (years)	61.5 ± 11.8	60.6 ± 16.4	0.90
Sex male	22 (79)	18 (64)	0.38
Body mass index (kg/m^2^)	23.6 [20.8–25.3]	23.3 [21.0–25.3]	0.89
ERI (IU/week/kg/(g/dL))	3.2 [2.0–5.8]	9.1 [7.7–12.7]	<0.01
Iron (µg/dL)	74.5 [56.5–96.0]	74.0 [50.3–94.8]	0.61
Transferrin saturation (%)	32.0 [21.5–40.1]	30.5 [21.3–37.4]	0.48
Ferritin (mg/mL)	141.9 [82.6–209.9]	138 [90.5–288.7]	0.53
C-reactive protein (mg/dL)	0.12 [0.05–0.34]	0.21 [0.04–0.58]	0.59
Corrected calcium (mg/dL)	9.0 ± 0.7	9.2 ± 0.6	0.32
Phosphorus (mg/dL)	5.5 ± 0.9	5.5 ± 1.4	0.71
Intact PTH (pg/dL)	273 ± 158	246 ± 147	0.52
Vitamin B12 (pg/mL)	558 ± 209	398 ± 216	0.13
Folate (ng/mL)	4.2 ± 2.1	6.1 ± 4.9	0.29
Albumin (g/dL)	3.4 ± 0.5	3.2 ± 0.4	0.18
Zinc (µg/dL)	59.5 [51.2–67.8]	62.0 [52.3–69.8]	0.64
Free carnitine (µmol/L)	29.8 [23.7–40.0]	24.4 [21.7–30.2]	0.03
Acyl carnitine/free carnitine ratio	0.5 [0.5–0.6]	0.6 [0.5–0.8]	<0.01

ERI: erythropoietin resistance index; PD: peritoneal dialysis; SD: standard deviation; IQR: interquartile range; Intact PTH: intact parathormone.

## Discussion

In this study, we found that carnitine deficiency was significantly correlated with the erythropoietin resistance index. To the best of our knowledge, this is the first report to show the correlation between carnitine deficiency and erythropoietin resistance in patients undergoing PD. In this study, 8% of patients who underwent PD had low free carnitine level (<20 μmol/L) and 93% of patients who underwent PD had a high acyl carnitine/free carnitine ratio (>0.4). Both of these indices are used for the definition of carnitine deficiency [7,8]. In our study, all patients with a low free carnitine level had a high acyl carnitine/free carnitine ratio. These results suggest that a high acylcarnitine/free carnitine ratio is more sensitive and has clinical utility in detecting carnitine deficiency compared with free carnitine level in patients undergoing PD. This could be because a high acylcarnitine/free carnitine ratio was independently associated with the erythropoietin resistance index in patients undergoing PD.

Several mechanisms can be considered for patients undergoing PD for developing carnitine deficiency. First, renal dysfunction leads to decreased biosynthesis and loss of preferential renal excretion of acyl carnitine [9,10]. Second, patients undergoing PD require restriction of foods containing carnitine owing to renal dysfunction [9,10]. Third, loss of free carnitine into PD fluid may also be involved [33]. Fourth, disproportionate clearance of carnitine before reaching the systemic circulation beyond the gastrointestinal compartment by PD fluid exchange may contribute to carnitine loss [34]. A previous study reported carnitine deficiency in infant patients undergoing PD [5], which is consistent with our results. Additionally, we found a significant correlation between carnitine deficiency and the PD period in univariate analysis. A previous study reported a high prevalence of carnitine deficiency in patients who underwent APD compared with those who underwent CAPD [35]. However, no correlations between the PD modality and carnitine deficiency were observed in our study. Further studies are required to investigate the correlation between PD modality and carnitine deficiency in patients undergoing PD.

Several studies have reported that carnitine deficiency is associated with anemia [2,36,37]. Carnitine contributes to stabilizing erythrocyte membranes, resulting in improvement of their deformability [2,36,37]. Previous studies have reported a significant correlation between carnitine deficiency and the erythropoietin resistance index, and improvement of erythropoiesis-resistant anemia by carnitine supplementation in patients undergoing hemodialysis [2,15,38]. Additionally, the erythropoietin resistance index is not only an indicator of erythropoietin-resistant renal anemia, but also a high mortality rate in patients undergoing hemodialysis [12]. Therefore, analyzing clinical factors associated with the erythropoietin resistance index in patients undergoing dialysis is important. Previous studies have reported that several factors, including iron deficiency, vitamin deficiency, chronic kidney disease-mineral and bone disorder (CKD-MBD), inflammation, and malnutrition are associated with erythropoietin resistance [23,39–52]. However, these associations were not observed in this study. This discrepancy between studies might be explained by the finding that the patients in our study had better control of levels of iron, transferrin saturation, ferritin, and vitamins (vitamin B12, folate), CKD-MBD, inflammation, and nutritional status compared with those in previous studies [23,39–52].

The National Kidney Foundation reported favorable effects of carnitine supplementation to patients undergoing hemodialysis [17]. The National Kidney Foundation recommends carnitine supplementation for 9 months to 1 year for erythropoietin-resistant anemia, hypotension during hemodialysis sessions, cardiac dysfunction, and muscle weakness [17]. However, carnitine supplementation for patients undergoing PD was not mentioned. A previous study reported showed that 12 patients who underwent PD showed improved erythropoietin-resistant anemia by carnitine supplementation [20]. However, another study reported that 12 infant patients who underwent PD did not have improved erythropoietin-resistant anemia by carnitine supplementation [19]. Additionally, the utility of using carnitine as an osmotic substance in PD fluid has been reported [53]. Furthermore, supplementation of carnitine by intravenous administration is more effective than oral carnitine intake in patients undergoing hemodialysis [54]. Another study showed that certain levels (80–199 μmol/L) of carnitine supplementation decreased clinical complications, such as hypotension and muscle cramps, during hemodialysis sessions compared with higher levels of free carnitine ( ≥ 300 μmol/L) in patients undergoing hemodialysis [6]. These results indicate that the supplementation route, monitoring of carnitine levels, and maintaining appropriate free carnitine levels by carnitine supplementation are important for decreasing clinical complications in patients undergoing hemodialysis and PD. Therefore, larger interventional studies are required to investigate the effects, including erythropoietin-resistant anemia, of carnitine supplementation for patients undergoing PD.

Our study has several limitations. First, because this study was conducted at a single institution, whether the results can be generalized at other institutions is unclear. Second, the design of this study was cross-sectional and observational. Therefore, we cannot suggest the importance of carnitine supplementation.

In conclusion, there is a high prevalence (93%) of carnitine deficiency (acyl carnitine/free carnitine ratio >0.4) in patients undergoing PD. Additionally, carnitine deficiency is significantly correlated with the erythropoietin resistance index. Clinicians should note this pathological condition in patients undergoing PD.
